# Developing mobile phone text messages for tobacco risk communication among college students: a mixed methods study

**DOI:** 10.1186/s12889-017-4027-z

**Published:** 2017-01-31

**Authors:** Alexander V. Prokhorov, Tamara C. Machado, Karen S. Calabro, Elizabeth A. Vanderwater, Damon J. Vidrine, Keryn P. Pasch, Salma K. Marani, Meredith Buchberg, Aditya Wagh, Sophia C. Russell, Katarzyna W. Czerniak, Gabrielle C. Botello, Mackenzie H. Dobbins, Georges E. Khalil, Cheryl L. Perry

**Affiliations:** 1 The University of Texas MD Anderson Cancer Center, 1155 Pressler Str., Unit 1330, Houston, TX 77030 USA; 2 Department of Behavioral Science, The University of Texas MD Anderson Cancer Center, 1155 Pressler Str., Unit 1330, Houston, TX 77030 USA; 3 Population Research Center, University of Texas at Austin, 305 E. 23rd Street, Austin, TX 78712 USA; 4 Department of Family and Preventive Medicine, The University of Oklahoma Health Sciences Center, 655 Research Parkway, Suite 400, Oklahoma City, OK 73104 USA; 5 Department of Kinesiology and Health Education, The University of Texas at Austin, 1 University Station, D 3700, Austin, TX 78712 USA; 6 Department of Periodontics, The University of Texas at San Antonio, 1 UTSA Circle, San Antonio, TX 78249 USA; 7 Michael & Susan Dell Center for Healthy Living, The University of Texas Health Science Center at Houston School of Public Health, 1616 Guadalupe St, Suite 6.300, Austin, TX 78701 USA

**Keywords:** Nicotine and tobacco products, Qualitative, Text messages, Young adults

## Abstract

**Background:**

Engaging young adults for the purpose of communicating health risks associated with nicotine and tobacco use can be challenging since they comprise a population heavily targeted with appealing marketing by the evolving tobacco industry. The Food and Drug Administration seeks novel ways to effectively communicate risks to warn about using these products. This paper describes the first step in developing a text messaging program delivered by smartphones that manipulate three messaging characteristics (i.e., depth, framing, and appeal).

**Methods:**

Perceptions of community college students were described after previewing text messages designed to inform about risks of using conventional and new tobacco products. Thirty-one tobacco users and nonusers, aged 18–25 participated in five focus discussions held on two community college campuses. Attendees reviewed prototype messages and contributed feedback about text message structure and content. Qualitative data were coded and analyzed using NVivo Version 10.

**Results:**

Most participants were female and two-thirds were ethnic minorities. A variety of conventional and new tobacco products in the past month were used by a third of participants. Three identified domains were derived from the qualitative data. These included perceived risks of using tobacco products, receptivity to message content, and logistical feedback regarding the future message campaign.

**Conclusion:**

Overall, participants found the messages to be interesting and appropriate. A gap in awareness of the risks of using new tobacco products was revealed. Feedback on the prototype messages was incorporated into message revisions. These findings provided preliminary confirmation that the forthcoming messaging program will be appealing to young adults.

## Background

Young adults are a dynamic population. Given the quickly evolving tobacco and nicotine markets, effective yet innovative approaches for communicating health information to this highly targeted population is crucial [1, 2]. Capturing the attention of young adults, however, can be challenging, especially if the objective is to communicate health risks associated with nicotine and tobacco use. Of the over 30 million 18- to 24-year-old Americans, about 20.2 million are enrolled in post-secondary institutions, providing a venue for testing tobacco control strategies among the young adult population [3, 4]. Between 2006 and 2014, current cigarette smoking decreased among college students from 19% to 12.9% [5]. This decline may be due to increasing popularity of new tobacco products [5]. Advertisements market these new products to young adults as safe alternatives to conventional tobacco products [6, 7]. Among the young adult populations, college students perceived the use of electronic cigarettes and hookah as favorable [8]. One nationally representative sample found that among 18- to 24-year-old cigarette smokers, 37.6% were exclusively cigarette smokers, while 62.4% were also users of other tobacco products (i.e., variants of cigars, smokeless products, hookah, and electronic cigarettes) [9]. Twenty-five percent of a national sample of young adults in 2014 smoked tobacco with a hookah. At 6-month follow-up, an additional 8% of never users initiated hookah [10]. Some young adults may be turning to alternative nicotine and tobacco products to decrease cigarette smoking, believing that they are reducing health risks associated with nicotine and tobacco use [11]. More research is needed to determine reasons for switching products, dual use and poly use; however, some attribute availability, absence of regulation, and an underestimation of the health risks as potential reasons [12, 13]. Identifying reasons for uptake and associated risks is an important endeavor in tobacco control for the young adult population.

The Family Smoking Prevention and Tobacco Control Act authorized the Federal Food and Drug Administration (FDA) to regulate conventional cigarettes and smokeless tobacco products 7 years ago [14]. Regulations were extended to electronic nicotine delivery systems (ENDS), cigars, and water pipe tobacco [15]. This act positions tobacco control research towards not only identifying constituents of addiction but also product preference, marketing appeal and health risk messaging. The current study documents the first step in the development of messages for a mobile phone text messaging campaign and the use of focus groups to test messages with the target audience. The campaign will consist of text messages designed to convey accurate risk information about conventional and new and emerging products. The majority of young adults (92%) in the United States own mobile devices, [16] thus delivering information via text messaging is a practical approach. Harnessing this commonly used form of communication provides an acceptable platform for informing the young adult population about health risks related to nicotine and tobacco use. It has the potential for closing gaps in health knowledge among difficult-to-reach populations [17, 18]. This mixed methods study describes the initial period for assessing the perceptions of a subset of the target population. Engaging in focus groups is an effective approach for determining perceptions [19]. Gauging the reactions of the targeted students to the drafted messages in focus groups was beneficial in guiding researchers to appropriately plan for writing the full inventory of messages.

## Methods

### Conceptualization and Development of Health Communication Campaigns

Development of the message campaign involved writing text messages designed to address health risks of nicotine and tobacco use. A multidisciplinary research team at the Texas Tobacco Center of Regulatory Science on Youth and Young Adults (Texas TCORS) composed the messages. Message development was guided by the Elaboration Likelihood Model (ELM) [20] and Prospect Theory [21]. Combined, these theories suggest message characteristics that shape message processing and ultimately increase comprehension, retention of message content and may shift attitudes. The ELM posits that complex and rational messages can incite *central processing* (i.e., cognitive processing, attention to message content, and opportunity for long-term attitude change). Simple and emotional messages can drive *peripheral processing* (i.e., non-cognitive affective processing, attention to the messenger and quality of the message, and opportunity for short-term attitude change) [20]. The Prospect Theory posits that individuals respond differently to a message, depending on whether it is framed in terms of costs (loss-framed) or benefits (gain-framed) [21]. Combining depth (i.e., simple or complex), appeal (i.e., emotional or rational) and framing (loss-framed or gain-framed) messages were structured into patterns of eight types of text messages (Table 1).

**Table 1:** Text message design and examples focused on hookah and electronic cigarettes (vaping)

Message type	Description	Message features
Depth		
Complex	Uses formal tone	Long sentences, scholarly vocabulary
Simple	Uses informal tone	Short, concise, language trends i.e. slang, popular text message abbreviations
Framing		
Gain	Conveys positive consequences of healthy behavior	Positive effects attained and negative effects that are reduced by avoiding products
Loss	Conveys negative consequences of unhealthy behavior	Negative effects when products are used
Appeal		
Emotional	Designed to evoke joy, fear, sarcasm, anger, sympathy, and humor around products	Emotional words, emotional context, and symbols representing either a smile or a frown
Rational	Presents logic and facts derived from scientific literature	Statements that use logic
Examples of Types of Messages	Hookah	Electronic Cigarettes
Simple/Loss/Rational	Water is not a filter. Ppl who smoke hookah are exposed to chemicals that are thought to be cleaned out by the pipe’s water.	Families who use e-juice put their toddler at risk since kids under 5 are most vulnerable to accidental e-juice poisoning.
Simple/Loss/Emotional	Ppl who smoke hookah have a higher chance of getting gross mouth herpes since they’re swapping germs thru mouthpieces. :(	Get this! The FDA can’t inform ppl of what’s in e-cigs. Ppl who vape lose control of what goes in their bodies. :( #WhatsInAVape
Simple/Gain/Emotional	Be free! Rejecting hookah helps ppl escape TWICE the amount of nicotine in a cig & the handcuffs of a crippling addiction! :)	Ppl who don’t use e-cigs keep little ones from making the tragic mistake of drinking e-cig juice & needing a terrifying trip to the ER. :)
Simple/Gain/Rational	Ppl who know the truth about hookah are safe from its smoke which has 36x more tar than a single cig.	Ever heard of e-juice & e-cigs? Staying away from e-cigs can lower the risk of becoming addicted since e-cigs are used to keep ppl craving nicotine.
Complex/Loss/Rational	People who socialize in hookah bars are more likely to experience respiratory conditions related to tobacco use.	Utilizing electronic cigarettes may lead to nicotine poisoning. Nicotine levels in refill cartridges can be inaccurately labeled with levels often higher than advertised.
Complex/Loss/Emotional	Letting your mouth write a check that your lungs cannot cash? College students frequenting hookah bars underestimate its damaging effects, leading to lung disorders. :(	Familiar with the liquid nicotine that fuels electronic cigarettes? Beware! Each drop specializes in dragging people into an abyss of addictive desire for these products! :(
Complex/Gain/Emotional	Germaphobe Jo has seen and smelled a fungus-covered toenail. Since she prefers fungus-free lungs, she avoids hookah pipes and the fungus that lurks within. :)	Avoid contamination! Electronic cigarettes are NOT regulated! Abstaining from vaping prevents breaches of potentially dangerous unknown substances into the body. :)

Abbreviations

*e-cigs* electronic cigarettes

*e-cig juice* liquid nicotine used to refill ENDS

*ppl* people

*cigs* cigarettes

### Recruitment

Participants were recruited from two Houston Community College campuses during the 2014–2015 academic year via flier postings and in-person recruitment. This system of colleges has about 69,293 students enrolled. The demographic profile of students was about 15% White, 13% Asian, 36% Hispanic, 32% African American with two thirds being from economically disadvantaged households [22]. The format for the sessions consisted of an online text message review and survey about the text messages. The qualitative items used in the discussion were standard concepts originating from the cognitive psychology and health education fields. Prior to use, a subset of college-aged individuals reviewed the items. They confirmed those aged 18 to 25 could comprehend the questions, reflect on the contents, and provide feedback. A self-administered traditional questionnaire captured demographics, tobacco use and risk perceptions, concluding with focus group discussions. There were 31 participants who engaged in all study aspects. Groups ranged from 5 to 10 people per group. The duration of the groups ranged from 1 to 2 h, depending on the size of the group and level of participation. One scheduled group had low attendance and resulted in an individual interview. A $25 gift card and refreshments were provided as compensation for participating. The study coordinator obtained written informed consent from each participant. The institutional review boards from The University of Texas MD Anderson Cancer Center and Houston Community College System approved this study.

### Data collection procedures

Participants reviewed a subset of 25 preliminary text messages via an online survey [23]. Participants rated the messages according to the depth, framing and appeal definitions as designated by researchers. Participants completed the self-administered survey capturing demographics and tobacco and nicotine use defined as past 30-day use. A risk perception measure was also included, generating discussion in response to “how much do you think people risk harming themselves (physically or in other ways) if they use the following tobacco products?” The 5-point rating scale responses were: no risk, slight risk, moderate risk, great risk, or can’t say [5]. Discussions began after the text message review, online survey and self-administered surveys. The study coordinator (T.C.M) and co-moderators (K.C., S.R.) conducted semi-structured discussions using key questions to learn reactions to and understanding of the messages by participants (Table 2) [24, 25].

**Table 2:** Key questions used during focus groups

As you read the messages, how confident did you feel in your ability to understand and to interpret them correctly?
How satisfying were the messages?
How about perceptions of the messages including: being interesting, well written, believable, persuasive, helpful, understandable, presented new information and coherent arguments?

In addition to key questions, discussions centered on appropriate message content, baseline knowledge of products, tolerance of survey questions, survey length and delivery logistics (time of day, frequency of messages). Each focus group was audio recorded and professionally transcribed.

### Data analysis

Descriptive statistics of participant characteristics and tobacco-related use were computed. We used QRS NVivo 10 software to code transcripts. The study coordinator (T.C.M.) and an additional coordinator (M.B.) served as coders and conducted ethnographic content analysis to identify common reported themes. A consensus procedure resolved coding discrepancies whereby both coders met weekly to compare text-derived domains and themes.

## Results

The majority of participants (67.7%) were female. The mean age was 22.4 years. The demographic distribution of participants was Hispanic (35.7%), Caucasian (33.3%), Asian (18.5%) and African American (48.1%) (Table 3). Two thirds did not indicate using tobacco. Overall, about 29% reported using tobacco and nicotine products (past 30-day use). One participant was an exclusive cigarette smoker; three participants exclusively used hookah. The remaining tobacco users were likely to smoke cigarettes concurrently with other products (Table 4). Risk perceptions of harm from cigarettes, chewing tobacco, and cigar use were greatest, ranging from 73% to 80%. Hookah and electronic cigarettes were perceived as the least harmful products. Several participants were unable to rate the health risks of the products as measured by the “can’t say” answer choice (Table 5).

**Table 3:** Demographic characteristics (*n* = 31)

Characteristic	Number	Percent
Gender		
Male	10	32.3
Female	21	67.7
Age in years	22.4 (mean)	2.3 (SD)
Ethnicity		
Hispanic/Latino	9	29.0
Not Hispanic/Latino	22	71.0
Race		
Caucasian	9	33.3
Asian	5	18.5
Black/African American	13	48.1

*SD* Standard deviation

**Table 4:** Use tobacco and nicotine products-past month use (*n* = 31)

Single product use	
Conventional cigarette only	1
Hookah only	3
Dual tobacco and nicotine product use	
Conventional cigarette, cigar	1
Conventional cigarette, hookah	1
Conventional cigarette, electronic cigarette	1
Electronic cigarette, hookah	2
Polytobacco and nicotine product use	
Little cigar, electronic cigarette, hookah	1
Conventional cigarette, pipe, various cigars	1
None	22

**Table 5:** Risk perceptions about tobacco nicotine and products (*n* = 31)

	No risk	Slight risk	Moderate	Great	Can’t Say
Product type	%	%	%	%	%
Cigarette	0.0	4.0	8.0	80.0	8.0
Dip, snuff	0.0	0.0	3.4	79.3	17.2
Chewing tobacco	0.0	0.0	3.6	78.6	14.3
Cigar	0.0	3.8	11.5	73.1	11.5
Pipe tobacco	3.4	3.4	10.3	65.5	17.2
Snus	0.0	7.1	3.6	60.7	28.6
Hookah	0.0	17.9	10.7	53.6	17.9
Little cigars, cigarillos, bidis	3.6	7.1	17.9	53.6	17.9
Electronic cigarettes	0.0	13.8	17.2	48.3	20.7

### Qualitative outcomes

Analyses of the qualitative data revealed three primary domains including: 1) perceived risk and product familiarity, 2) receptivity to message content, and 3) logistical feedback. The major themes that emerged from the discussions are described within each of the domains described below. The first major theme involved perceived risk of using tobacco/nicotine products. The majority of responses were related to perceived risk about hookah, electronic cigarettes and personal vaporizers. Participants expressed their beliefs and beliefs thought to be held by peers. They described marketing strategies on television, radio and across social media platforms that included enticing and positive imagery. They elaborated about the positive effects of use, especially for electronic cigarettes. Participants reported that positive media portrayal has convinced many that new products are less harmful compared to conventional products. One participant reported:

*“… e-cigarettes have been on the news saying how beneficial they are or at least…more beneficial than if you’re actually smoking….” (Male, focus group 3102)*

In discussions, participants associated hookah with low risk. They believed that the social setting of hookah use contributes to the less harmful image. They also believe that the social aspects of hookah use leads to peer pressure to use. One person reported:

*“…hookah… you know lounges and stuff like that…it seems like this fun, social thing, and like it’s sort of framed in a much different context from like smoking cigarettes…”(Male, focus group 3103)*

Some described flavoring as enticing and associated with lower perceived risk leading to acceptance and experimental use:

*“…hookah…it just makes people feel more like you’re part of the group because everyone passes it around… And honestly…it doesn’t taste that bad. That and the vaporizers, the e-cigs… you can taste the flavors, and that’s it.” (Female, Focus group 3106)*

In general, participants described emerging products, particularly e-cigarettes and hookah, as less harmful and more socially acceptable due to positive marketing and enticing flavors that lead to experimentation.

The second major theme was product familiarity. Participants spoke about tobacco products that they had personally used, products they had seen friends and family members use, media portrayal, and observations of product promotions such as newly developed lounges, shops and advertisement. Hookah and electronic cigarettes were the most familiar emerging products among participants. Some comments included the following:

*“Everywhere you go there’s a hookah shop somewhere.” (Female, focus group 3105)*

*“…there used to be an old store that sold like grocery products. And then all of a sudden, the next week, it’s a smoke shop or a hookah lounge…it’s gaining in popularity…” (Female, focus group 3106)*

In general, snus was the least familiar product. This was followed by liquid nicotine, a product used in electronic cigarettes and personal vaporizers. While participants were familiar with electronic cigarettes, many were not aware that a liquid was used in the devices. Many were not aware that liquid nicotine could be purchased and used as a standalone product. In addition, many were not aware that tobacco and nicotine are present in hookah and electronic cigarettes.

A third important theme was marijuana use. In one of the groups, participants requested educational information on the dual use of cigars and marijuana, a substance use behavior known as “blunting.” Blunting is a term used when the tobacco of a cigar is removed and replaced with marijuana. The tobacco leaves of the cigar are then re-rolled, with the marijuana replacing the tobacco inside of the cigar. This new product (cigar filled with marijuana) is referred to as a “blunt.” The discussion centered on the presence of nicotine in a blunted cigar. The moderator informed the group about the presence of nicotine in the cigar’s tobacco leaves themselves. Many of the participants were not aware that tobacco and nicotine still existed in the blunted cigar wrapper and stated that this new information will likely deter their use. As one participant asked:

*“So you’re still smoking nicotine when you’re smoking marijuana?” (Female, focus group 3105)*

Participants also provided feedback on the messages themselves. Overall reception was positive. Participants described the messages as informative, interesting, easy to understand, straight to the point, and at an appropriate character limit for a health-related text message. In one of the focus groups, students expressed a desire for more information about emerging products verses conventional products:

*“You do not want to use it even more…It increases you ‘no’ to ‘H-no.’” (Female, focus group 3105)*

Messages that described the risk of changed appearance due to use, such as discolored teeth and increased risk of premature aging were often referenced in the discussions. In all groups, participants expressed that they particularly liked the messages about appearance which forewarned about the possibility of negative effects from tobacco use, such as skin aging and bad breath. Participants felt that these types of messages would strongly resonate with their peers. As one participant noted:

*“And nowadays I feel like people care about their looks and no one doesn’t want anything that’s going to mess it up.” (Female, focus group 3105)*

In response to messages constructed with an emotional appeal, one participant stated:

*“Yeah, the emotional ones were persuasive because people like to keep up appearances… but the ones that gave out facts, I didn’t really feel like it was persuading. It was just telling me, matter of fact, “This is what it is.” (Female, focus group 3106)*

Messages about health and disease were also frequently mentioned. Participants specifically referenced messages about chronic obstructive pulmonary disease, asthma, diabetes and limb amputation during the discussion.

During the focus groups, participants were presented with the delivery of the health communication campaign scheduled for the main study. This was described as having two 30-day campaigns consisting of two text messages per day and various assessments accessible via their personal smartphone. Participants commented that this campaign would provide an appropriate dose. They made a recommendation about timing to ensuring the health communication campaign would be well received. This was to send the messages at a time of day that community college students would be most likely to read them (10 am and 7 pm).

## Discussion

The described research efforts are crucial for developing and testing public health messages that are not only scientifically valid but are also believable, convincing and well-received by young adults. The role of the FDA is to regulate products and communicate risks to protect public health [26]. By extension, the National Institutes of Health granted the Texas TCORS with the significant and stimulating task of expertly crafting health communication campaigns for the young adult population.

Our participants revealed a gap in awareness of the risks of using new tobacco products, particularly for hookah and electronic cigarettes. All were familiar with the range of products and their popularity among peers. Discussions revealed limited knowledge about the products. These qualitative findings support the results on the self-administered survey. This sample found the text messages as conveying accurate information about the risks of tobacco use as interesting and appropriate. These findings provide preliminary confirmation that our forthcoming health campaign using health statistics and risk information extracted from the current literature, coupled with pop culture language in the messages, will be well-positioned to appeal to young adults. Participant feedback was evaluated by researchers that resulted in appropriate modifications to the messages, study materials, and future plans for campaign deployment.

Progress on the message campaign has advanced to completion of 972 text messages. We are concluding the vetting of the entire text message library by reviewers that include a group of experts from communication and tobacco control and university students enrolled in communication programs. Reviewers are rating each message for depth, framing and appeal using a Likert scale response format. The aim is to confirm group consensus regarding agreement among reviewers of the message structure as intended by researchers. This will be followed by a longitudinal randomized study among 640 community college students. Participants will receive one of eight message types, each representing a unique combination of characteristics based on a 2^3^ factorial design. We will identify the most potent of eight message combinations for conventional vs. new tobacco products.

### Strengths and limitations

The strength of this study is that it provided an opportunity to introduce a diverse group of students attending the targeted campuses to the preliminary work on the health campaign. It is also one of the first manuscripts to examine alternative tobacco products within a community college setting [27]. Findings regarding poly use of tobacco and lower risk perceptions for hookah and electronic cigarettes were similar to other reports among young adults and college students [9, 28–30]. The NVivo 10 software was useful in the qualitative analysis since it helped to identify new domains. A limitation was this was a sample of students from a limited geographic area, however and did not represent the entire campus. Finally, applied to this text messaging library, we acknowledge one size does not always fit all, and that over time, the text messages will not remain the same. Our design of the text messages is only a first step. The sample size of 31 participants is supported by previous qualitative research. Previous research recommends four groups of focus group discussions [31, 32]. Also, for the focus groups, the organization of participants in groups of 5 to 10 is recommended by previous research [31–34]. After the trial, the library of text messages will continue to be refined and become tailored to specific generations and sociodemographic and cultural groups. When the text messages are used for specific contexts such as a specific sociodemographic group, we do plan to work with the intended population in order to refine the text messages.

## Conclusions

In conclusion, this mixed methods study provided valuable insight during the health campaign development stage regarding favorable acceptance, interest in the topic, and deficits in knowledge about health risks of the products. We anticipate that the results of the campaign will augment efforts by the FDA to fulfill the priority mission of educating the public with effective and evidence-based risk communication strategies. Results can inform professionals who work with a diverse, low income population of young adults about strategies that accurately present the risks of nicotine and tobacco products. This information, in turn, is intended to result in a decline in consumption of tobacco products and tobacco-related disease.

## Abbreviations

Cigs: Cigarettes
e-cig juice: Liquid nicotine used to refill ENDS
e-cigs: Electronic cigarettes
ELM: Elaboration Likelihood Model
ENDS: Electronic nicotine delivery systems
FDA: Food and Drug Administration
Ppl: People
TCORS: Tobacco Centers of Regulatory Science

## References

[1] Cortese DK, Lewis MJ, Ling PM (2009). Tobacco industry lifestyle magazines targeted to young adults. J Adolesc Health.

[2] Biener L, Albers AB (2004). Young adults: vulnerable new targets of tobacco marketing. Am J Public Health.

[3] US Census Bureau, Population Division: Annual Estimates of the Resident Population for Selected Age Groups by Sex for the United States, States, Counties, and Puerto Rico Commonwealth and Municipios: 2015. April 1, 2010 to July 1, 2014. https://www.census.gov/popest/data/datasets.html. Accessed 25 Sept 2016.

[4] U.S. Department of Education, Institute of Education Sciences, National Center for Education Statistics: Digest of Education Statistics. 2013. http://nces.ed.gov/programs/digest/. Accessed 25 Sept 2016.

[5] Johnston L, O’Malley P, Bachman J, Schulenberg J, Meich R (2015). Monitoring the Future: National survey on drug use 1975–2014 college students and adults ages 19–55.

[6] Sterling KL, Fryer CS, Majeed B, Duong MM (2015). Promotion of waterpipe tobacco use, its variants and accessories in young adult newspapers: a content analysis of message portrayal. Health Educ Res.

[7] Grana RA, Ling PM (2014). “Smoking revolution”: a content analysis of electronic cigarette retail websites. Am J Prev Med.

[8] Berg CJ, Stratton E, Schauer GL, Lewis M, Wang Y, Windle M, Kegler M (2015). Perceived harm, addictiveness, and social acceptability of tobacco products and marijuana among young adults: marijuana, hookah, and electronic cigarettes win. Subst Use Misuse.

[9] Lee YO, Hebert CJ, Nonnemaker JM, Kim AE (2014). Multiple tobacco product use among adults in the United States: Cigarettes, cigars, electronic cigarettes, hookah, smokeless tobacco, and snus. Prev Med.

[10] Villanti AC, Cobb CO, Cohn AM, Williams VF, Rath JM (2015). Correlates of hookah use and predictors of hookah trial in U.S. young adults. Am J Prev Med.

[11] Gottlieb JC, Cohen LM, Haslam AK (2014). Comparing college smokers’ and dual users’ expectancies towards cigarette smoking. Addict Behav.

[12] Goodwin RD, Grinberg A, Shapiro J, Keith D, McNeil MP, Taha F, Jiang B, Hart CL (2014). Hookah Use Among College Students: Prevalence, Drug Use, and Mental Health. Drug Alcohol Depend.

[13] Butler KM, Ickes MJ, Rayens MK, Wiggins AT, Hahn EJ (2016). Polytobacco Use Among College Students. Nicotine Tob Res.

[14] Family Smoking Prevention and Tobacco Control Act. http://www.govtrack.us/congress/bill/. Accessed 25 Sept 2016.

[15] Food and Drug Administration: Extending Authorities to All Tobacco Products, Including E-Cigarettes, Cigars, and Hookah. 2016. http://www.fda.gov/TobaccoProducts/Labeling/. Accessed 25 Sept 2016.

[16] Pew Research Center: 2016 Smartphone usage continues to climb. http://www.pewglobal.org/2016/02/22/smartphone-ownership. Accessed 25 Sept 2016.

[17] Hospital MM, Wagner EF, Morris SL, Sawant M, Siqueira LM, Soumah M (2016). Developing an SMS Intervention for the Prevention of Underage Drinking: Results From Focus Groups. Subst Use Misuse.

[18] Suffoletto B, Kristan J, Callaway C, Kim KH, Chung T, Monti PM, Clark DB (2014). A text message alcohol intervention for young adult emergency department patients: a randomized clinical trial. Ann Emerg Med.

[19] Krueger RA, Casey MA (2014). Focus groups: A practical guide for applied research.

[20] Petty RE, Cacioppo JT (1986). The Elaboration Likelihood Model of Persuasion. Communication and Persuasion: Central and Peripheral Routes to Attitude Change.

[21] Tversky A, Kahneman D (1981). The framing of decisions and the psychology of choice. Science.

[22] Houston Community College, Office of Institutional Research: Houston Community College 2015–2016 Fact Book. http://www.hccs.edu/district/about-us/oir/hcc-fact-book/2015-2016-Fact-Book.pdf. 2016. Accessed 25 Sept 2016.

[23] Qualtrics Software. https://www.qualtrics.com/. 2016: Accessed 25 Sept 2016.

[24] Holbrook MB. Beyond attitude structure: Toward the informational determinants of attitude. J Market Res. 1978;15:545–56.

[25] Vidrine JI, Simmons VN, Brandon TH (2007). Construction of Smoking‐Relevant Risk Perceptions Among College Students: The Influence of Need for Cognition and Message Content. J Appl Soc Psychol.

[26] Department of Health and Human Services, US Food and Drug Administration, Office of the Commissioner, Office of the Chief Scientist: Advancing Regulatory Science for Public Health. http://www.fda.gov/ScienceResearch/SpecialTopics/RegulatoryScience/ucm228131.htm: 2010: Accessed 25 Sept 2016.

[27] Montgomery SB, De Borba-Silva M, Singh P, Dos Santos H, Job JS, Brink TL (2015). Exploring Demographic and Substance Use Correlates of Hookah Use in a Sample of Southern California Community College Students. Calif J Health Promot.

[28] Latimer LA, Batanova M, Loukas A (2014). Prevalence and harm perceptions of various tobacco products among college students. Nicotine Tob Res.

[29] Doran N, Brikmanis K (2016). Expectancies for and use of e-cigarettes and hookah among young adult non-daily smokers. Addict Behav.

[30] Wackowski OA, Delnevo CD (2016). Young Adults’ Risk Perceptions of Various Tobacco Products Relative to Cigarettes: Results From the National Young Adult Health Survey. Health Educ Behav.

[31] Kitzinger J (1996). Introducing focus groups in qualitative research.

[32] Twinn D (1998). An analysis of the effectiveness of focus groups as a method of qualitative data collection with Chinese populations in nursing research. J Adv Nurs.

[33] Krueger RA, Casey MA. Focus groups: A practical guide for applied research. Thousand Oaks: Sage; 2015.

[34] Langford BE, Schoenfeld G, Izzo G (2002). Nominal grouping sessions vs focus groups. Qual Mark Res Int J.

